# Biomarkers in Multiple Sclerosis: An Up-to-Date Overview

**DOI:** 10.1155/2013/340508

**Published:** 2013-01-22

**Authors:** Serafeim Katsavos, Maria Anagnostouli

**Affiliations:** Immunogenetics Laboratory, 1st Department of Neurology, Aeginition Hospital, National and Kapodistrian University of Athens, 115 28 Athens, Greece

## Abstract

During the last decades, the effort of establishing satisfactory biomarkers for multiple sclerosis has been proven to be very difficult, due to the clinical and pathophysiological complexities of the disease. Recent knowledge acquired in the domains of genomics-immunogenetics and neuroimmunology, as well as the evolution in neuroimaging, has provided a whole new list of biomarkers. This variety, though, leads inevitably to confusion in the effort of decision making concerning strategic and individualized therapeutics. In this paper, our primary goal is to provide the reader with a list of the most important characteristics that a biomarker must possess in order to be considered as reliable. Additionally, up-to-date biomarkers are further divided into three subgroups, genetic-immunogenetic, laboratorial, and imaging. The most important representatives of each category are presented in the text and for the first time in a summarizing workable table, in a critical way, estimating their diagnostic potential and their efficacy to correlate with phenotypical expression, neuroinflammation, neurodegeneration, disability, and therapeutical response. Special attention is given to the “gold standards” of each category, like HLA-DRB1∗ polymorphisms, oligoclonal bands, vitamin D, and conventional and nonconventional imaging techniques. Moreover, not adequately established but quite promising, recently characterized biomarkers, like TOB-1 polymorphisms, are further discussed.

## 1. Introduction

Multiple Sclerosis (MS) is the most common reason of neurological disability among young adults. Its clinical course varies greatly, reflecting complexity in pathophysiology. Different mechanisms of inflammation-demyelination, axonal damage-neurodegeneration, gliosis, and remyelination-repair combine together in various degrees (influenced by idiosyncratic factors) to create a unique clinical result for each patient. Identifying those idiosyncratic factors, as well as understanding which mechanism is prominent in each case, is the first step towards a rational therapeutical choice. Thus, guiding research towards distinguishing reliable biomarkers for every independent MS pathogenic factor is of primary importance.

An adequate definition of the term “biomarker” would be as follows: “Biomarker is a characteristic that is objectively measured and evaluated as an indicator of normal biological processes, pathogenic processes or pharmacological responses to a therapeutical intervention” [1]. A biomarker would ideally serve as a “surrogate endpoint” of a clinical outcome, only if it was fully capable to represent it. At this time, such a measure exists only in a theoretical basis, because none of the existing biomarkers can fully reflect the immensity of diverse MS pathogenic mechanisms creating a unique result. Arguably, the applicability of a biomarker for MS should be judged by the use of certain criteria. The criteria that were used in the present paper, in accordance with previous efforts of systematization [2, 3], and are reflected in a critical way in Table 1, are the following:

- *biological rationale*: degree of the correlation of the biomarker with an identified pathogenic mechanism;
- *clinical rationale*: accuracy in depicting clinical status;
- *predictability* of disease initiation, reactivation, or progression, or of disease differentiation of other demyelinating spectrum diseases, like NeuroMyelitis Optica (NMO);
- *sensitivity and specificity*: false negative or false positive results in depicting an event;
- *reproducibility* of a result;
- *practicality* of the method in use for the measurement;
- *correlation with therapeutic outcome*: reflecting the negative and positive effects of a therapy;
- *correlation with prognosis and disability*: the latter being objectively measured by instruments such as the Expanded Disability Status Scale (EDSS).

## 2. Materials and Methods

### 2.1. Cerebrospinal Fluid (CSF)

Its value as a means of providing biomarkers is undebatable, due to its natural proximity to the Central Nervous System (CNS). The levels of a CSF biomarker cannot be influenced by liver or kidney function.

On the other hand, the invasiveness of the collecting method narrows the potential of multiple measurements. Circadian fluctuation in CSF's production rate dictates the necessity of standardizing the time of performing a lumbar puncture [4]. Little is known about circadian fluctuation in the concentrations of CSF biomarkers. Experience acquired in other fields of neurological science suggests that this may be the case (i.e., circadian fluctuation in the levels of hypocretin-1, dopamine, and tryptophan) [5–8]. It is hypothesized, although not clarified in all papers, that CSF collection via lumbar puncture is done in morning hours, after night fasting.

### 2.2. Blood

It is easier to collect compared to CSF, with fewer limitations regarding safety. There is also a limitation concerning circadian fluctuations. Interleukin-(IL-)6 has maximum concentration at 08:00 a.m., and minimum at 22:00 p.m. [9]. Tumor Necrosis Factor (TNF) Receptor-1 and Receptor-2, soluble Intercellular Adhesion Molecule-1 (sICAM-1), and soluble Vascular Cell Adhesion Molecule-1 (sVCAM-1) also display diurnal variations [10]. Kidney function, liver function, and concomitant infections can influence the levels measured, as well as the time from collection to process.

### 2.3. Urine

It is the easiest material to collect, even in a 24-hour basis, overcoming the obstacles of fluctuations previously mentioned. Bacterial colonization of the urinary tract though can distort the measurements. MS patients with bladder dysfunction may regulate the amount of the fluids taken in a daily basis, affecting the quantity of produced urine.

### 2.4. Tears

There have been previous efforts in measuring OligoClonal Bands (OCBs) in tears, with results comparable to those of CSF [11].

### 2.5. Saliva

It has served as a means of specifying soluble Human Leucocyte Antigens (HLAs) type II [12].

### 2.6. Imaging Methods

Magnetic Resonance Imaging (MRI) was considered, till recently, as the most accurate imaging method for MS. There are though considerable difficulties in correlating MRI findings with disability progression. Certain novel ambitious techniques promise to overcome all these problems. The most important of them are further analyzed in paragraph 7. 

## 3. Measuring Techniques

Although in depth analysis of all the different techniques providing biomarkers for MS is out of the scopes of this paper, a short comment in relevance to the most important of them is going to follow. An example biomarker will be provided in each case, extracted from the literature and references sited in this paper. 

### 3.1. Enzyme-Linked Immunosorbent Assay (ELISA)

It is the most common method for specifying soluble proteins. The term refers to a solid-phase enzyme immunoassay which gives the ability to detect the presence of a substance, in a liquid or wet sample. Performing an ELISA technique requires the use of at least one specific antibody against the antigen under investigation. There are numerous different commercial kits available. By the use of standardization protocols, the assays are easy to reproduce by different laboratories. ELISA techniques give also the opportunity to analyse samples that are properly stored (i.e., IL-1 and IL-6 measurements; see Section 6.1.8).

### 3.2. Immunofluorescence 

This is a highly sensitive technique which gives the opportunity to specify, in vivo, proteins of variable size, haptens, and antibodies. Immunofluorescence techniques use the affinity of a certain antibody against its epitope, which is made visible through microscope by the use of proper fluorescent dyes. Further categorization in direct and indirect methods refers to the use of one or two antibodies. Time-resolved immunofluorescence assays display high sensibility, tracking down proteins in very small concentrations. Like ELISA, this technique is highly reproducible and accessible by different laboratories. Major drawback is the danger of photobleaching, as a result of prolonged light exposure (i.e., sICAM-1 and sVCAM-1; see Section 6.1.9)

### 3.3. Flow Cytometry

It is a laser-based method applied in the study of cellular subpopulations and biomarkers by the form of surface antigens, DNA and RNA variations, protein expression, enzymes, and intracellular antigens. Several techniques, like fluorescein dyes and intracellular cytokine dying, allow the collection of functional data for specific cellular populations. The major drawback of these assays is that sample handling may influence the results. Because of that, comparisons between different biomarker measurements are at risk of being inaccurate (i.e., *α*4 integrin; see Section 6.3.1)

### 3.4. Polymerase Chain Reaction (PCR)

PCR provides the researcher with the ability to detect trivial amount of genetic information. Basic components of the method are primers (short DNA fragments, sequences complementary to the target area), as well as a DNA polymerase, which enhances the replication. Quantitative PCR methods include competitive, noncompetitive, and real-time PCR. Posttranslational modifications are not taken into account by PCR techniques. Subsequently, the results may not be fully compatible with the functional state, in vivo. Cost and sample handling limitations should also be considered (i.e., HLA-DRB1* polymorphisms; see Section 5.1)

### 3.5. Nephelometry

The term nephelometry refers to a technique of estimating protein concentrations in different bodily fluids. The liquid sample is beamed by light by a certain angle, and afterwards the degree of light scatter is estimated. Nephelometry techniques are widely performed, reliably reproducible by many different laboratories (i.e., CSF albumin; see Section 6.4)

### 3.6. Western Blotting

The term refers to a protein detection method, which uses gel electrophoresis of a sample and subsequent dying of the protein target by the use of a specific antibody, on a membranic surface. Several final detection techniques have been developed, namely, colorimetric and fluorescent (i.e., *α*B-Crystalline measurements; see Section 6.5.2).

### 3.7. Isoelectric Focusing

This is another electrophoresis technique, which takes advantage of the phenomenon of the different isoelectric point between different molecules, in order to separate them. For this purpose, acrylamide gels with pH gradient are used (i.e., detection of CSF oligoclonal bands-kappa and lambda free chains; see Sections 6.1.1, 6.1.2 and 6.1.3) [13].

### 3.8. “-Omics” Technologies

The general term “-omics” refers to a group of rapidly emerging novel technologies that give the opportunity of large-scale analysis and identification of candidate biomarkers in multiple levels of cell biology (DNA, RNA, proteins, lipids, metabolites, and epigenetic modifications). Subsequently, the “-omics” technologies are further categorized in:

- genomics: large-scale studies of the whole DNA sequence (i.e., vitamin D Receptor Element recognition; see Section 6.2.2);
- transcriptomics: genome-wide studies of RNA sequences. Two main types of transcriptomics technologies are in common use, microarrays and next generation sequencing (i.e., TOB-1 gene downregulation; see Section 5.3) [14];
- proteomics: large-scale studies of protein distribution (i.e., Ninjurin-1; see Section 6.4.2);
- lipidomics: recognition studies of important cellular lipid pathways. Recent knowledge implicates specific CNS lipid epitopes in the generation of anti-lipid antibodies in MS (see also IgM against myelin lipids Section 6.1.2) [15];
- metabolomics: studies of important metabolic pathways, as a result of MS specific pathogenic mechanisms (i.e., N-acetyloaspartate CSF measurements; see Section 6.8.6);
- epigenomics: large-scale studies of epigenetic modifications. They explore the potential influence that alterations in chromatin architecture may have in MS susceptibility. One such study reported similar methylation profiles between twins discordant for MS [16].

## 4. Classifications

In this paper biomarkers are further categorized in three subgroups for reasons of systematic approach, and according to their pathophysiological implication in MS pathogenesis:

- genetic/immunogenetic: biomarkers specified via genomics and immunogenetic techniques;
- laboratorial: all other biomarkers that can be measured in body fluids;
- imaging: biomarkers provided by imaging techniques.

## 5. Genetic-Immunogenetic Biomarkers

The fact that genetic factors may influence MS was already known by epidemiological studies of previous decades. Recent research, using modern techniques previously mentioned, led to the implication of multiple genetic loci, with polymorphisms of Major Histocompatibility Complex (MHC) antigens having the primary role. 

### 5.1. HLA

#### 5.1.1. Genetic Risk

 Polymorphisms in HLA class II antigens seem to be decisive in attributing genetic burden for MS. Initial studies found positive correlation between DRB1*1501-DRB5*0101-DQA1*0102-DQB1*0602 haplotype and disease frequency. Multiple recent researches, conducted in many MS cohorts, made clear that HLA-DRB1*1501 is the mainly responsible allele for attributing genetic risk in MS population [17–21]. Moreover, HLA-DRB1*1501 expression is partially regulated by vitamin D through interaction in a genomic level, thus explaining the long known connection between latitude and risk for MS (see also Section 6.2.2). Additionally, coexistence of certain alleles probably leads to augmentation of the overall risk, via epistatic mechanisms (i.e., DRB1*1501 and DQ1*0102) [22]. Nevertheless, there have been reports of association with other HLA loci in some studies, like DR3 and DR4 haplotypes in Sardinia [23] and DRB1*04 in Hutterite families [24], as a mere reflection of the disease's genetic complexity. A positive association of the haplotype DRB1*1303-DQA1*05-DQB1*030 with MS was found in the non-Ashkenazi Jewish population [21]. Finally, HLA-DQ *και* HLA-DP polymorphisms were found to correlate with opticospinal MS frequency in Japanese cohorts [25].

#### 5.1.2. HLA and OCB

Positive correlation of HLA-DRB1*1501 and negative correlation of HLA-DRB1*0405 alleles with OCB in the CSF of MS patients were established by observations in a Japanese cohort [26] and further confirmed by subsequent studies.

#### 5.1.3. Clinical and Imaging Correlations

HLA-DRB1*15 was found to correlate positively with early onset MS [27]. Its correlation with early progression from relapsing-remitting MS (RRMS) to secondary progressive MS (SPMS) remains controversial [28, 29]. HLA-DRB1*01 allele is considered to protect against malignant disease form [30]. 

Zivadinov et al. observed the following in a study of MS patients [31]:

- DRB1*1501 positive had worst brain atrophy scores and bigger T1 lesions' burden in MRI;
- DQB1*0301 positive had worst brain atrophy scores and bigger T2 lesions' burden;
- DQB1*0602 positive had worst grey matter atrophy scores.

In another study [32], DRB1*1501 positive patients had the following:

- lower *N*-acetyl-aspartate (NAA) levels within normal appearing white matter (NAWM);
- bigger white matter lesions;
- worst brain atrophy scores;
- impaired cognitive function.

#### 5.1.4. HLA and Therapeutical Choice

HLA-DRB1*0401, 0408, and 1601 alleles correlate with greater risk of developing neutralizing antibodies against interferon beta (IFN-*β*), resulting in poor therapeutical outcome [33].

### 5.2. Non-MHC Polymorphisms Attributing Genetic Risk

Various genome-wide studies revealed many non-MHC single nucleotide polymorphisms as candidates for genetic burden augmentation in MS. Most of them though had only a modest effect on susceptibility. Polymorphisms of the IL2RA and IL7RA regions seem as the most promising at the moment [34]. Increasing evidence also implicates other loci, like EVI5, CD58, KIAA0350, and RPL5 genes [35]. Another recent genome-wide association study identified 57 non-MHC genes associated with MS [36].

### 5.3. TOB-1

TOB-1 gene has a role against T-cell multiplication, keeping autoreactive cells in a dormant state. Its degreased expression leads towards a more intense immune response (higher percentage of Th1 and Th17 cells and lower percentage of T-regulatory cells). TOB-1 polymorphisms represent an independent factor influencing the progression from clinically isolated syndrome (CIS) to clinically definite multiple sclerosis (CDMS) [37].

### 5.4. Apolipoprotein E (ApoE)

ApoE is a protein regulating lipid homeostasis, located mostly in astrocytes. Carrying *ε*4 allele of ApoE seems to attribute greater risk of developing mental disorders in MS patients [38].

## 6. Laboratorial Biomarkers

### 6.1. Biomarkers of Immunological Activation

#### 6.1.1. OCB IgG in CSF

Positive OCB IgG in the CSF of patients with CIS was found to duplicate the risk of progression in CDMS in a 4-year observation period [39]. Additional studies provide more evidence for OCB IgG being a relevant factor for conversion to CDMS [40]. Their diagnostic sensitivity is high (>90%), but they lack in specificity (~35%) among inflammatory disorders of the CNS. OCB IgG could not be connected with known protein targets inside CNS, like myelin basic protein (MBP), proteolipid Protein, and myelin oligodendrocyte glycoprotein (MOG) [41].

#### 6.1.2. OCB IgM in CSF

Some researchers consider them as a bad prognostic biomarker, correlating with disability progression both qualitatively and quantitatively (IgM index) [42]. In contrast, other studies could not confirm these findings [43]. OCB IgM against certain myelin lipids may declare a more aggressive disease course [44]. 

#### 6.1.3. Kappa Free (KFLC) and Lambda Free Light Chains (LFLC) in CSF

KFLC high CSF levels have been repeatedly reported in MS. In comparison to OCB IgG, slightly higher sensitivity with slightly lower specificity has been found [45]. KFLC high CSF levels are considered as highly predictive for CIS conversion to CDMS [46]. LFLC also represent a sensitive indicator of intrathecal synthesis in inflammatory CNS disorders [47].

#### 6.1.4. Measles-Rubella-Zoster Endothecal Reaction (MRZ Reaction)

MRZ IgG reaction in CSF displays, compared to OCB IgG, a higher specificity for MS diagnosis and higher prognostic value of progression from CIS to CDMS [48]. Moreover, MRZ reaction indicates a primarily B-cell mediated immune response, guiding therapeutical choice towards a relevant immunomodulating agent [49].

#### 6.1.5. Epstein-Barr Virus (EBV) Reaction

Cepok et al. reported a high percentage of IgG antibodies against protein epitopes BRRF2 and EBNA-1 of the virus, in the serum and CSF samples from MS patients [50]. Lünemann et al. isolated from MS patients in 2006 [51] highly specific T-cells for the epitope EBNA-1. Recently, infection of blood-brain barrier (BBB) endothelial cells has been reported, initiating disruption mechanisms, as well as proinflammatory cytokines production [52]. EBV antibodies are considered as indicative of higher inflammatory activity and early disease onset [53]. 

#### 6.1.6. Antibodies against MBP and against MOG

Their diagnostic and prognostic values in MS remain highly controversial. Initially, they were regarded as satisfactory predictors of conversion from CIS to CDMS [54]. Other studies though did not reach the same conclusions [55]. Considerable part of the controversy is actually attributed to the initial methodological diversity. Specifically, it has been made clear that the anti-MOG antibodies recognize only conformational epitopes of MOG and not their linear counterparts displayed in soluble phase [56, 57]. Likewise, denaturation of MBP leads to a marked decrease in IgM antibody induced in vitro deposition [58]. Nevertheless, occurrence of anti-myelin antibodies alone could not support a diagnosis of increased risk for future MS, in the preclinical stage of the disease [59].

Considerably higher levels of serum autoantibodies against the fragments 48–70 and 85–170 of MBP were found in MS patients compared to healthy controls [60]. Reactivity against fragments 43–68 and 146–170 was also found significantly different between MS patients and donors suffering from other neurological disorders (ONDs). Naturally occurring MBP autoantibodies in healthy controls may have diminished capacity of facilitating interferon gamma (IFN-*γ*) and IL-5, than those occurring in MS [58]. Presence of anti-MBP antibodies in childhood MS increases the risk of an acute demyelinating encephalomyelitis-(ADEM-) like onset of the disease [61].

 Finally, children with CIS were found to have high titres of anti-MOG in a percentage of 30%–40% [62]. In general, anti-MOG antibodies are considered to play significant role in childhood MS, anti-Aquaporine 4 (AQP4) negative NMO, ADEM, and isolated optic neuritis, but hardly in adult MS [63, 64]. Some markers of intrathecal anti-MOG production though, like the rMOG index, may have some use in adult MS, especially in progressive disease forms [65].

#### 6.1.7. Chemokines

Chemokine CXCL13 mobilizes B-cells and T-helper cells towards active demyelinating lesions by interacting with CXCR5 receptor. Consistent correlation of CXCL13 CSF levels with CSF B-cells, plasmablasts, and intrathecal Ig synthesis has been reported [66]. High levels of CXCL13 have been found in patients with CIS and CDMS [67]. On the other hand, chemokine CXCL12 was shown to have a protective role against CNS inflammation, in experimental autoimmune encephalomyelitis (EAE). Nevertheless, data still remain experimental [68]. CCL2 levels, normally induced by Th2 immune response, were found diminished shortly after methylprednizolone treatment for MS relapse, but not one month later [69].

#### 6.1.8. Cytokines

Inflammatory activity in active demyelinating lesions leads to the liberation of many different cytokines that can be used as biomarkers of disease activity. Proinflammatory cytokines in the periphery primarily originate from T- and B-cells, whereas B-cells seem to be mainly responsible for their intrathecal production in RRMS [70], with monocytes having a more immunoregulatory role inside CSF. IFN-*γ* and TNF-a are the main products of Th1 immune response. IL-6 serves as linking arm between B-cell and T-cell immune response as well as a Th-17 response triggering factor. IL-6 serum levels were found to correlate significantly with the relapse frequency in female MS patients and age at onset for all MS patients [71].

Moreover, studying IL-1 levels in mice led to the conclusion that any imbalance in the IL-1 signalling (increased or decreased) may lead to CNS demyelination [72]. IL-10 is considered as the main anti-inflammatory cytokine. Recent research implicates single nucleotide polymorphisms at the −592 position of the IL-10 gene to the regulation of CNS autoimmunity [73]. Flow cytometric analysis revealed that B-cells and monocytes from MS patients overexpress IL-15, and that stimulation of CD8(+) T-cells with the latter cytokine enhances their ability to kill glial cells and enter the BBB [74]. IL-15 was found elevated in the sera and CSF of MS patients, in comparison with ONDs [75]. Finally, treating EAE mice with anti-IL-33 led to decreased levels of IFN-*γ* and IL-17 and upregulation of proinflammatory IL-10 and transforming growth factor-*β* (TGF-*β*) [76].

#### 6.1.9. Adhesion Molecules

Proinflammatory cytokines cause a rise in CSF expression of sICAMs. High levels of ICAM-1 molecule correlate positively with higher disease activity [77]. Higher CSF levels of sICAM-1 and sVCAM-1 were reported in NMO patients, in comparison with MS patients, suggesting that the BBB in NMO displays more severe alterations [78]. Finally, laminin 411, which is situated within the vascular endothelium, interacts with adhesion molecule CD146, allowing Th17 cells to overcome the BBB [79].

#### 6.1.10. Osteopontin

Osteopontin is a macrophage derived phosphoprotein which enhances IFN-*γ* and IL-12 levels and diminishes the levels of neuroprotective IL-10. Serum and CSF osteopontin levels are upregulated during an MS relapse, but this is also the case for many other inflammatory disorders [80]. CSF CCR2(+)CCR5(+) T-cells show a distinct ability to produce osteopontin during relapse [81].

#### 6.1.11. Fetuin-A

Fetuin-A (alpha2 Hermans Schmid glycoprotein) is a calcium-regulating surface glycoprotein. Protein's coding m-RNA is overexpressed in MS patients' CNS, resulting in its high concentrations in active demyelinating lesions. Fetuin-A seems to antagonize anti-inflammatory TGF-*β*1. Good responders in Natalizumab treatment present a reduction in Fetuin-A CSF levels [82].

### 6.2. Biomarkers of Neuroprotection

#### 6.2.1. Vascular Endothelial Growth Factor-A (VEGF-A)

VEGF-A is a factor of angiogenesis with neuroprotective properties. Diminished m-RNA expression of VEGF-A in serum monocytes of patients with SPMS compared to RRMS patients has been reported. VEGF-A could serve as biomarker of progression from RRMS to SPMS [83].

#### 6.2.2. Vitamin D

The vitamin's potential pathogenic role in MS can be deducted by multiple previous epidemiological studies that showed correlation of latitude and sun exposure with relative risk for developing the disease. Vitamin D suppresses Th1 immune response in multiple levels and enables the production of many neurotrophic factors. 25-Hydroxyvitamin D levels in untreated MS patients correlate inversely with radiologic disease activity [84]. Recently, a vitamin D response element (VDRE) was recognized close to the HLA-DRB1*1501 coding area, with the aid of genomics. Vitamin D displays an inhibitory role in MS, also at a genetic level, by interacting with VDRE [85].

Interestingly, Stewart et al. recently concluded that part of IFN-*β* therapeutic effects during MS relapses may be attributed to greater production of Vitamin D [86].

#### 6.2.3. Other Vitamins as Biomarkers—Pathogenetic Factors in MS

Evidence from animal model research also implicates vitamins other than vitamin D in MS pathogenesis. Fat-soluble vitamins A and E are considered as modulators of disease activity [87]. Moreover, a small series of patients with MS found decreased CSF and serum levels of the vitamin biotin, in comparison to healthy controls [88].

### 6.3. Biomarkers of Cellular Subpopulations

#### 6.3.1. B-Cells

Mature B-cells and plasma-blasts were found to accumulate in the CSF of RRMS patients, correlating positively with higher disease activity in the MRI [89]. Centroblasts, typically found in the germinal centre of lymphatic tissue, were also detected in the CSF of MS patients, suggesting intrathecal production [90]. Transitional alterations of B-cells in early MS stages, like overexpression of CD80(+), *α*4, and *β*1 integrins, are associated with their ability to cross BBB [91]. The expression of *α*4 integrin in B-cells was correlated with the number of gadolinium-enhanced lesions, during CIS [92].

#### 6.3.2. T-Cells

Autoreactive memory T-cells enter CNS with the help of CXCR3 cytokine receptor. CXCR3 has a low diagnostic specificity for MS, due to its high levels in many other inflammatory disorders [93]. Recent studies revealed expansion of CD4(+)CD28(−) T-cells in the sera of MS patients, T-cells phenotype with enhanced cytotoxic properties. They were described to accumulate in CNS lesions, following a fractalkine gradient. CX(3)CR1, the fractalkine receptor, may serve as biomarker for discriminating CD4(+)CD28(−) cells from their CD28(+) counterparts [94]. CSF CCR2(+)CCR5(+) T-cells show remarkable increase during MS relapse, which is not the case in ONDs, displaying reactivity against MBP [81].

#### 6.3.3. Natural Killers (NK) Cells

RRMS patients in a remission phase display high levels of CD-56 surface antigen in their NK cells (>36%). CD56 bright NK cells may regulate T-cell survival in MS [95]. 

#### 6.3.4. T-Cell Receptor Excision Circles (TRECs)

TRECs are intracellular by-products of T-cell receptor remodelling, gradually rejected via homeostatic mechanisms. Existence of TRECs inside a T-cell is a good marker of naivety. Thymic gland's functional state can be estimated by the percentage of naïve T-cells in the peripheral blood. A definite reduction of TRECs in MS patients has been reported, indicative of thymic dysfunction in the disease. Naïve T-cells are found further diminished in patients with primary progressive multiple sclerosis (PPMS), compared to RRMS [96].

#### 6.3.5. Lipocalin 2

Lipocalins are a family of proteins that transport small hydrophobic molecules, taking part in several processes of the immune system. The gene encoding for lipocalin 2 was found upregulated during relapses in the EAE model of MS, mainly originating from neutrophils infiltrating the Choroid Plexus (CP), as well as from astrocytes in affected regions. CSF levels of lipocalin 2 were found increased in two different MS cohorts. Decrease, subsequent to clinical response after Natalizumab treatment, was also reported [97].

### 6.4. Biomarkers of Blood-Brain Barrier Disruption

BBB disruption is an early feature of lesion formation, leading to edema, excitotoxicity, and entry of serum proteins and inflammatory cells inside CNS. Intercellular endothelial tight junctions breakdown possesses a primary role between events leading to BBB and blood-cerebrospinal fluid barrier (BCB) disruption [98, 99]. Tight junction proteins occluding and claudin-1 showed decrease after treatment of a BBB model with MBP [100]. 

Apart from CSF production, CP is actually considered as important regulator of CNS autoimmunity, displaying properties of early BCB disruption site, allowing sentinel T-cells to enter noninflamed regions [101, 102]. Expression of various adhesion molecules in the vascular endothelium plays a vital role in facilitating inflammatory agents from the periphery to enter the barrier (see also Section 6.1.9). 

Various measures of BBB permeability have been proposed, like the CSF : serum albumin ratio (AR). AR levels are constantly higher in NMO in comparison to MS, and display correlation with clinical severity only in NMO [103].

#### 6.4.1. Matrix Mettalloproteinase Proteins (MMPs)

Serum and CSF MMPs levels are constantly elevated during MS relapse. MMP-9 levels have been found elevated in patients with RRMS [104]. CSF CCR2(+)CCR5(+)CCR6(−) T-cell population expresses high levels of MMP-9 during relapse [81].

#### 6.4.2. Ninjurin-1

The degree of expression of the protein Ninjurin-1 by endothelial cells of the BBB and myeloid antigen-presenting cells (APCs) plays an important role in the transmigration and localization of the latter inside CNS, as it was made obvious by proteomic screen of human BBB cells. Ninjurin-1 was found up-regulated in active demyelinating lesions [105].

#### 6.4.3. sICAM-1

The CSF sICAM-1 levels from NMO patients were found to correlate adequately with other measures of BBB disruption, like the albumin quotient and the gadolinium-enhanced lesions in MRI [78]. (see also Section 6.1.9).

#### 6.4.4. Endothelin System

The term refers to an endothelial proteinic system that plays role in the transmigration of monocytes through the BBB. Major components of this system are the proteins endothelin-1, endothelin type B receptor, and endothelin-converting enzyme-1 [106].

#### 6.4.5. EBV Infection

Further information about EVB infection is in Section 6.1.5.

### 6.5. Demyelination Biomarkers

#### 6.5.1. Myelin Basic Protein

MBP and its fragments are found in large quantities in the CSF of most MS patients during a relapse (80%) [107]. High concentrations can also be found though in many ONDs. A significant correlation of decrease in CSF-MBP, contrast-enhancement in MRI, and clinical disability in response to treatment with methylprednisolone suggests an association between inflammation and myelin breakdown in MS [108].

#### 6.5.2. *α*B-Crystalline

Immunohistochemical analysis of demyelinating lesions revealed increased expression of this protein, comparatively to healthy myelin. *α*B-Crystalline is a heat-shock protein which forms aggregates during stress. It is considered as primary target molecule for T-cells in MS, but it can also be found elevated in the CSF of patients with ONDs [109]. Its mechanism of action encompasses activation of IL-17, IL-10, IL-13, TNF, and chemokines CCL5 and CCL1 [110].

### 6.6. Biomarker of Oxidative Stress

#### 6.6.1. Nitric Oxide (NO) and Its Metabolites

NO and its metabolites can cause mitochondrial damage and tissue hypoxia leading to further damage in MS lesions. High serum and CSF levels of NO in inflammatory neurological disorders were reported. Higher CSF concentrations were further correlated with higher disability progression rates in MS [111].

#### 6.6.2. Reactive Oxygen Species (ROS)

ROS damage oligodendrocytes and myelin through radical mediated oxidation. Myelin cholesterol breaks down to 7-ketocholesterol, whose levels in the CSF of MS patients have been reported to be elevated [112].

### 6.7. Excitotoxicity Biomarkers

#### 6.7.1. Glutamate

Extracellular levels of glutamate are normally regulated through its active reabsorption in oligodendrocytes. In active demyelinating lesions, homeostatic mechanisms are distorted resulting in extracellular glutamate accumulation that causes further axonal damage [113]. Active lesions seem to overexpress cystine/glutamate antiporter, aiming in intracellular accumulation of cystine for production of the antioxidant glutathione [114].

### 6.8. Biomarkers of Axonal Damage

#### 6.8.1. Neurofilaments (NFs)

Neurofilaments are major axonal cytoskeleton proteins consisting of three subunits (light chain/NF-L, intermediate chain/NF-M, and heavy chain/NF-H). NF-L CSF levels in MS patients are considerably higher compared to healthy controls and ONDs patients, reaching their peak approximately three weeks after relapse onset [115]. Their correlation, however, with disability progression is poor. CSF NF-L levels in Natalizumab responders have been reported to return to normal [116].

On the other hand, NF-H chains seem to correlate better with disease progression, with significant elevation recorded only in progressive disease forms [117].

#### 6.8.2. Tau Protein

Tau is a cytoskeleton protein whose basic responsibility is microtubular stabilization. High CSF levels in MS patients have been reported. Simultaneous elevation in Tau and NF-H values in CSF, in patients with CIS, has a 70% predictive value of conversion to CDMS, which is superior to the predictive value of MRI [118].

#### 6.8.3. Microtubules

Microtubules represent a major structural cytoskeleton component, consisting of two subunits, A- and B-tubulin. They are closely associated with tau protein and microfilaments, especially actin. Elevated CSF tubulin and actin values have been reported in progressive disease forms, correlating well with disability measured by EDSS [119].

#### 6.8.4. Amyloid-*β* (1–42)

In Alzheimer's disease, amyloid-*β* (1–42) accumulates in extracellular insoluble plaques, resulting in reduced CSF levels. CSF reduction can be also observed in MS patients, in correlation with greater risk for cognitive decline [120].

#### 6.8.5. 14-3-3 Protein

Apart from Creutzfeldt-Jacobs disease, elevated CSF values have been reported in 10%–30% of patients with RRMS [119], but its potential utility as a biomarker for MS seems limited for the time being. 

#### 6.8.6. N-AcetyloAspartate (NAA)

NAA is an aminoacid, highly expressed in neurons, which transfers actively water molecules extracellularly against concentration gradient. Spectroscopy techniques revealed decreased NAA values in MS lesions, but also in NAWM, in conventional MRI. CSF-NAA reduction correlates adequately with disability progression [121]. 

On the contrary, serum and CSF NAA levels were significantly higher in RRMS patients, in comparison to healthy donors and NMO patients. Subsequently, NAA could be helpful in differential diagnosis between MS and NMO [122].

### 6.9. Biomarkers of Glial Activation Dysfunction

#### 6.9.1. S100B Protein

S100B is a calcium-binding protein, primarily expressed in astrocytes, whose CSF elevated values have been previously correlated with cerebral injury. There are reports of CSF elevation in RRMS patients [123], but overall data remain inconclusive. 

#### 6.9.2. Glial Fibrillary Acidic Protein (GFAP)

GFAP is a structural protein of the astrocytes whose CSF levels increase in association with gliosis-astrocytosis. High CSF values have been found in SPMS patients, but rarely in RRMS patients, and seem to correlate well with disability progression [115, 116]. CSF-GFAP levels are significantly higher during NMO relapse, in comparison with MS relapse [124–126], and show adequate correlation with clinical improvement and disability progression in NMO [125, 126]. S100B possesses the same properties, but correlations tend to be weaker [125, 126].

### 6.10. Biomarkers of Remyelination Repair

#### 6.10.1. Neuronal Cell Adhesion Molecule (N-CAM)

Constant CSF elevation of N-CAM has been repeatedly reported immediately after MS relapse, in adequate correlation with clinical improvement. N-CAM is assumed to have a key role in remyelination process. The exact pathway still remains unclear [127].

#### 6.10.2. Brain-Derived Neurotrophic Factor (BDNF)

Lower CSF-BNDF levels in SPMS patients comparatively to RRMS patients have been reported. Low BDNF levels are considered to contribute in demyelination and axonal damage progress [128]. BDNF increased production was observed in Glatiramer Acetate responders, correlating well with clinical improvement [129].

#### 6.10.3. Soluble Molecule Nogo-A

Nogo-A is a CNS myelin component that inhibits axonal repair. Its presence in MS patients CSF constitutes a bad prognostic marker of axonal repair. Nogo-A is adequately specific for MS, as it could not be isolated in other autoimmune or infectious neurological disorders [130].

### 6.11. Biomarkers of Therapeutical Response

#### 6.11.1. Interferon Beta **(**IFN-*β* **)**

IFN-*β* converts peripheral immune response from the Th1 towards the more anti-inflammatory Th2, influencing the expression of many genes. Many ways of predicting treatment effects with IFN-*β* have been proposed. HLA-DRB1 polymorphisms influence neutralizing antibodies production (Section 5.1.4). TNF-related inducing ligand (TRAIL) and Myxovirus Resistance Protein-A (MxA) serum levels may also reflect response to IFN-*β* [131]. Finally, upregulation of sVCAM levels, following IFN-*β* treatment, may represent a reliable predictor of therapeutical response [132].

Progress in understanding of MS pathophysiology shed light over the important role of Th17 immune response. Recently, researchers reached the conclusion that MS patients with prominent Th17 response are probably more harmed than benefited by treatment with IFN-*β*, because of IL-17 exacerbation by the drug [133]. Nevertheless, IL-17F levels could not be connected with poor therapeutic outcome during IFN-*β* treatment by other researchers [134].

Vitamin D levels during  IFN-*β* treatment are mentioned in Section 6.2.2.

#### 6.11.2. Glatiramer Acetate

Predictive role of BDNF is mentioned in Section 6.10.2.

#### 6.11.3. Natalizumab

Expression levels of unblocked *α*4 integrin on peripheral mononuclear blood cells can serve as biomarker of Natalizumab therapeutical efficacy, as well as biomarker of risk for progressive multifocal leucoencephalopathy (PML) [135]. Predictive role of Fetuin-A is mentioned in Section 6.1.11, predictive role of lipocalin 2 is mentioned in Section 6.3.5, and predictive role of NF-L is mentioned in Section 6.8.1.

#### 6.11.4. Cladribine

CSF levels reduction of sICAM-1 and sE-Selectin may potentially serve as biomarkers of therapeutical efficacy after cladribine treatment [136].

## 7. Imaging Biomarkers

### 7.1. Optical Coherence Tomography (OCT)

OCT is a noninvasive technique using emission of infrared light through the pupil and detection of its reflection from the retina. Retinal nerve fiber layer (RNFL) thickness can then be estimated. RNFL thinning can be used as a reliable biomarker of axonal loss, correlating adequately with brain atrophy measures [137]. RNFL thickness can serve as biomarker of disease progression and neuroprotection by a certain therapeutical agent [138].

### 7.2. Magnetic Resonance Imaging (MRI)

MRI provides the clinical doctor with a substantial variety of neuroinflammation biomarkers. On the other hand, classical MRI techniques lack in adequate correlation with neurodegeneration and disability progression.

The most important MRI biomarkers for MS are the following:

- T1 lesions with contrast enhancement: biomarkers of acute neuroinflammation. Although they are considered as the gold standard for BBB disruption imaging, recent research claims that the same diagnosis can be inferred in many cases by combination of T1, T2, and T2-weighted FLAIR images characteristics alone [139];
- hyperintense T2-weighted lesions: reflecting a combination of mechanisms like inflammation, demyelination, axonal damage and edema. Their diagnostic value is high, but they correlate moderately with disability [140];
- hypointense T1-weighted lesions (black holes): considered as satisfactory biomarkers of axonal damage [141]. Their correlation with disability remains debatable [142];
- whole brain atrophy biomarkers: the most widely used measure is the brain parenchymal fraction. Brain atrophy worsening rates are higher in untreated MS patients (0.5%–1% annualized decrease) in comparison with healthy controls (0.1%–0.3%) [143]. Brain atrophy worsening rate at initial diagnosis has been proposed as prognostic biomarker of disability eight years afterwards [144];
- gray matter atrophy biomarkers: recently acquired knowledge suggests gray matter demyelination, axonal damage, and atrophy in MS. Double inversion recovery imaging techniques display gray matter atrophy in all MS stages and types, with higher worsening rates in SPMS patients [145]. Higher worsening rates of gray matter atrophy in CIS patients correlate well with rapid conversion to RRMS [146];
- spinal cord atrophy biomarkers: upper cervical cord area (UCCA) measuring techniques display atrophy most apparently in progressive MS forms, correlating well with disability progression. UCCA atrophy presence in early disease stages in RRMS patients is a bad prognostic biomarker of future disability [147].

### 7.3. Contrast Magnetization Transfer Ratio (MTR)

It is a novel MRI technique based on proton interaction between free water and macromolecules. In the absence of axonal loss, acute MRI lesions that show recovery display also increase in MTR [148]. Optic nerve MTR decrease after optic neuritis shows good correlation with RNFL thickness in OCT (Section 7.1) and with reduction of amplitude in visual evoked potentials, suggesting that MTR is primarily an axonal damage biomarker [149]. Nevertheless, reliable assessment of treatment effects on remyelination has been reported [150].

### 7.4. Diffusion Weighted Imaging (DWI) and Diffusion Tensor Imaging (DTI)

DWI is based on mobility and spatial distribution of water molecules, while DTI measures movement in several directions in space. DTI technique provides two different measures, mean diffusivity (MD) and fractional anisotropy (FA).

MD increases and FA decreases in hyperintense T2-weighted lesions. Similar alterations can be recorded in NAWM areas in conventional MRI, as well as in normal appearing gray matter (NAGM) areas, especially in progressive disease forms [151]. Corpus callosum DTI abnormalities are present in early MS stages, even when lesions in conventional MRI are still absent [152]. MD alterations precede visible in conventional MRI BBB injury by at least 5 months, being thus a reliable predictive biomarker for MS relapse [153]. Corpus callosum DTI abnormalities in SPMS patients constitute a bad prognostic biomarker of future disability [154]. 

### 7.5. Magnetic Resonance Spectroscopy (MRS)

MRS is a novel imaging method for assessment of pathobiochemical disease processes. The following substances spectroscopic measurements are of particular value in MS:

- NAA: biomarker of neuronal and axonal integrity. NAA showed a progressive decline pattern in a two-year MRS followup of patients with RRMS [155];
- choline: biomarker of myelin loss;
- myoinositol and creatine: biomarkers of gliosis;
- glutamate: biomarker of acute inflammation.

Early spectroscopic changes represent a bad prognostic factor of future disability [156]. Spectroscopic findings suggest that white matter abnormalities in RRMS are more prominent than grey matter abnormalities where the injury is less diffuse [157].

Diffusion tensor spectroscopy (DTS), a technique combining properties of DTI and MRS, seems promising in better distinguishing axonopathy, demyelination, inflammation, edema, and gliosis [158].

### 7.6. Positron Emission Tomography (PET)

Modern PET tracers have the ability to bind in proteins that show upregulation in activated microglia, making possible an early visualization of NAWM and NAGM disorders, even before contrast enhancement in conventional MRI [159]. At present, the use of PET in MS remains experimental.

### 7.7. Evoked Potentials (EPs)

EPs estimate action potential conduction along somatosensory, motor, visual, and auditory pathways, providing a reliable means of demyelination and axonal loss assessment. MRI techniques have diminished their spectrum of use, although visual and motor EPs may have some utility as biomarkers of neurodegeneration [160]. Motor EPs are of special value in patients with MS presentation as a spinal cord syndrome [161]. Sensory EPs in combination with EDSS score could help in predicting short-term progression of disability in MS [162]. Vestibular evoked myogenic potentials (VEMPs) can offer complementary information in relevance with brainstem dysfunction [163]. 

Combined EP data, like the EP score, for instance, could offer a reliable prognostic biomarker, especially for early recognition of benign MS forms [164]. Prediction of disease course over a three-year period has been reported [165].

### 7.8. Sympathetic Skin Response (SSR)

Recent studies of SSR in MS patients report correlation of SSR abnormalities with higher EDSS scores and disease duration. SSR may be a useful tool of autonomic function assessment among MS patients [166].

## 8. Conclusions

Pathophysiological complexity of MS leads inevitably to a great variety of potential biomarkers, as it was made obvious by the previous analysis. Thus, for systematization reasons and after the completion of an elaborate quest for biomarkers in the international literature we

1. subgrouped all biomarkers in six broad categories, that is, diagnostic biomarkers, biomarkers of phenotypical expression, biomarkers of demyelination—neuroinflammation—relapse, biomarkers of axonal loss—neurodegeneration, prognostic biomarkers—biomarkers of disability progression, and biomarkers of therapeutical response;
2. gradually evaluated every biomarker with (+++) to (+/−) according to their implication in the category they referred to;
3. commented on biomarker abilities to reflect the properties of the category in which they are presented.

The previous three steps demanded as prerequisite hard, critical, and thorough data processing, in order to achieve the best and accurate results, since it is the first time that an attempt of such a systematization is done in a workable table. In this table, biomarkers such as HLA-II alleles and OCBs are for many years the “gold standard” for MS, while other well-described biomarkers are being implicated more and more every day, like conventional and nonconventional MRI scans. Additionally, many other laboratorial and imaging parameters are at the beginning of their characterization as biomarkers in MS.

From every group of biomarkers, we collected those with characterization of (+++) and presented them as an easy summary to the reader, as it follows. Additionally, we present the biomarkers of differentiation between MS and NMO:

- *MS diagnosis*: the panel of potential biomarkers should definitely include HLA-DRB1* characterization, CSF OCB IgG and/or KFLC, CSF MRZ reaction, serum vitamin D levels, MRI with contrast-enhancement, and EPs;
- *MS phenotypical expression*: the panel of potential biomarkers should primarily include HLA-DRB1* characterization, antibodies against confrontational epitopes of MBP and MOG, rMOG index, and IL-6 serum levels. DTI abnormalities and UCCA atrophy measures may also be helpful;
- *demyelination-neuroinflammation-relapse*: the panel of potential biomarkers should definitely include contrast-enhanced T1 MRI lesions, CSF MBP-Glutamate-NF-L and CCR2(+)CCR5(+) T-cells, serum levels of IFN-*γ*-TNF-*α*-IL-6-vitamin D, and expression of TOB-1-Fetuin-A-*α*B-crystalline. AR, CSF sICAM-1, and CSF sVCAM-1 are good indicators of BBB disruption. MRS offers reliable data in the study of demyelinating lesions;
- *axonal loss-neurodegeneration*: NAA and Nogo-A have the leading position as potential biomarkers. RNFL and T1 black holes offer also valuable information. DTS may help even more in the near future;
- *prognosis-disability progression*: OCB IgG, KFLC, MRZ reaction, combination of NF-H with tau, TOB-1 expression, and higher worsening rates of gray-matter atrophy are reliable prognostic biomarkers of CIS conversion to CDMS. NAA, NF-H, and combined EP's reflect adequately disability progression in MS, and GFAP does the same in NMO. DTI abnormalities offer prognostic data for relapse and disability progression;
- *therapeutical response*: HLA-DRB1* polymorphisms and vitamin D levels should be considered as biomarkers for therapeutical outcome for IFN-B. Fetuin-A, NF-L, and unblocked *α*4 integrin represent biomarkers for Natalizumab treatment and BDNF for glatiramer acetate treatment. RNFL may be offered substantially in the near future;
- *differentiation from NMO*: MRS findings should help when differential diagnosis is required. CSF NAA, CSF GFAP, and AR can also help when there is a possibility for NMO (apart from antiAQP4 of course).

Even after decades of research, MS still remains at a significant proportion an unsolved mystery. This is mainly the reason why finding a biomarker with absolute surrogacy abilities remains elusive. Further research in the field of MS biomarkers must be directed towards an earlier and accurate diagnosis and a more prompt, targeted, and individualized therapeutical approach, with the minimum intervention and economical cost.

## Figures and Tables

**Table 1:** Biomarkers in MS.

(A) Diagnostic biomarkers (criteria i, iv, v, and vi)		
(1) Genetic-immunogenetic		
HLA-DRB1*1501	+++ Risk for MS	See also B, E
DR3 and DR4 haplotypes	++ Risk for MS	
HLA-DRB1*04	++ Risk for MS	
HLA-DRB1*0401	+ Risk for high familial autoimmunity in MS patients	See also F
HLA-DQ1*0102	+ Risk for MS, in coexistence with HLA-DRB1*1501	
HLA-DPB1*0501	+ Risk for opticospinal MS	
HLA-DPB1*0301	+ Risk for opticospinal MS	
IL2RA and IL7RA polymorphisms	+ Risk for MS	
EVI5, CD58, KIAA0350, and RPL5 polymorphisms	+/− Risk for MS	
(2) Laboratorial		
OCB IgG	+++ But with low specificity	See also E
KFLC	+++ But with low specificity	See also E
MRZ reaction	+++ Higher specificity than OCB IgG	See also E, F
Anti BRRF2, anti EBNA-1	++	See also B, C
Anti MBP 48–70 and 85–170	+	See also B, E
Anti MBP 43–68 and 146–170	+ Differential diagnosis with OND's	See also B, E
MBP/MOG conformational epitopes antibodies	+ But low specificity	See also B, E, F
VEGF-A	+ Lower CSF levels in all disease forms, but low specificity	See also D, E
Vitamin D	+++ Lower levels, higher risk for MS	See also C, F
TRECs	+ Lower serum levels in all disease forms, but low specificity	See also B
CSF levels of lipocalin 2	+ Higher CSF levels in MS, but low specificity	See also F
AR	+++ Differential diagnosis of MS and NMO	See also C, E
NO and NO metabolites	+ Higher CSF and serum levels in MS, but low specificity	See also C, E
NF-L	++ Higher CSF levels in MS patients	See also C, F
NAA	+++ Differential diagnosis of RRMS and NMO	See also D, E
GFAP	+++ Differential diagnosis of MS and NMO	See also C, E
S100B	+ Differential diagnosis of MS and NMO	See also C, E
Nogo-A	++ For MS forms with prominent neurodegenerative element	See also D
(3) Imaging		
Contrast-enhanced T1 lesions	+++	See also C
Hyperintense T2-weighted lesions	+++	See also C, D, E
Corpus callosum DTI abnormalities	++ Early diagnostic biomarker	See also E
MRS findings (glutamate/choline)	+++	See also C, D, E
PET	++ But still experimental	
EPs Motor EPs VEMPs	+++ +++ Spinal cord syndrome at presentation +++ Brainstem dysfunction	See also C, D, E
SSR	++ Autonomic dysfunction assessment in MS patients	See also E

(B) Biomarkers of phenotypical expression (criteria ii, iv, v, and vi)		

(1) Genetic-immunogenetic		
HLA-DRB1*1501	+++ Early disease onset	See also A, E
HLA-DRB1*1501	+ Risk for cognitive decline	
HLA-DRB1*01	++ Protection against malignant disease form	
ApoE *ε*4	++ Greater risk for mental disorders	
(2) Laboratorial		
OCB IgM against myelin lipids	+/− Aggressive disease course	See also E
EBV antibodies	+ Early disease onset	See also A, C
Anti-MBP	+++ ADEM-like onset in childhood MS	See also A, E
Anti-MOG	+++ Childhood MS, ADEM, isolated optic neuritis, anti-AQP4 (−) NMO	See also A, E, F
rMOG index	+++ Progressive disease forms	
IL-6 serum levels	+++ Age at onset	See also C
TRECs	++ Lower levels PPMS	See also A
Amyloid-*β* (1–42)	++ Lower levels, higher risk for mental disorders	
(3) Imaging		
UCCA atrophy	+++ Progressive disease forms	See also E
NAGM DTI abnormalities	+++ Progressive disease forms	

(C) Biomarkers of demyelination-neuroinflammation-relapse (criteria i, ii, iii, iv, v, and vi)		

(1) Genetic-immunogenetic		
TOB1	+++ Underexpression, higher Th1 and Th17 percentage	See also E
(2) Laboratorial		
EBV antibodies	+ Higher inflammatory activity	See also A, B
CXCL13	++ Mobilizes B-cells, T-helper cells	
CXCL12	+/− Neuroprotection against inflammation in EAE/ experimental	
IFN-*γ*/TNF-a	+++ Th1 immune response	
IL-1 levels imbalance	+ Triggering factor for neuroinflammation	
IL-6	+++ B-cell and T-cell immunity link, Th17 immune response triggering factor ++ Correlation with relapse frequency in female MS patients	See also B
IL-10 −592 position polymorphisms	++ Regulation of CNS autoimmunity	
IL-15	++ BBB disruption, enhanced CD8(+) T cytotoxicity	
IL-33	+ Increase in IFN-*γ* and IL-17 in mice EAE	
sICAM-1	++ Higher levels, higher inflammatory activity +++ Higher levels in NMO than MS—marker of BBB disruption	See also F
sVCAM-1	+++ Higher levels in NMO than MS—marker of BBB disruption	See also F
Laminin 411	++ TH-17 enhancement	
*α*4 Integrin	++ Correlation with gadolinium-enhanced lesions during CIS	See also E, F
Osteopontin	++ Serum and CSF elevation during relapse	
Fetuin-A	+++ Overexpression in active demyelinating lesions	See also F
Vitamin D	+++ High levels, anti-inflammatory role—lower radiological disease activity	See also A, F
CSF mature B-cells/plasma-blasts	++ Bigger accumulation, higher inflammatory activity	
CXCR3	++ Helps T-cells to enter the brain	
CX(3)CR1	++ CD4(+)CD28(−) cytotoxic cells biomarker	
CSF CCR2(+)CCR5(+) T cells	+++ Increase during MS relapse—osteopontin enhancement	
CD56 Bright NK	++ Remission phase	
AR	+++ Biomarker of BBB disruption	See also A, E
MMP-9	++ Higher CSF levels during relapse	
Ninjurin-1	++ Upregulation in active demyelinating lesions	
MBP and fragments	+++ Higher CSF levels during relapse	See also F
*α*B-Crystalline	+++ Over-expression in active demyelinating lesions	
NO and metabolites	++	See also A, E
7-Ketocholesterol	++	
Glutamate	+++ Higher levels in active demyelinating lesions	
Cystine/glutamate antiporter	+ Over-expression in active demyelinating lesions	
NF-L	+++ Higher CSF levels, especially the 3rd week after relapse onset	See also A, F
GFAP	++ Higher levels during relapse	See also A, E
S100B	+/− Higher CSF levels during MS/NMO relapse	See also A, E
N-CAM	+ CSF elevation at remission onset	
BDNF	++ Lower levels inhibit demyelination and axonal loss	See also D, E, F
(3) Imaging		
Contrast-enhanced T1 lesions	+++ Active lesions	See also A
Hyperintense T2-weighted lesions	++ Combination of different mechanisms	See also A, D, E
MTR decrease	+ Demyelination and axonal loss combined	See also D
DTI abnormalities	++ Combination of different mechanisms	See also D, E
MRS findings (especially changes in glutamate and choline)	+++ Active lesions	See also A, D, E
DTS	++ Promising but still experimental	See also D
EP's delayed conduction	++ Demyelination biomarker	See also A, D, E

(D) Biomarkers of axonal loss-neurodegeneration (criteria i, iv, v, and vi)		

(1) Laboratorial		
VEGF-A	++ Lower levels, higher risk for neurodegeneration	See also A, E
14-3-3	+/− Axonal loss	
NAA	+++ Axonal loss	See also A, E
BDNF	++ Lower levels inhibit demyelination and axonal loss	See also C, E, F
Nogo-A	+++ Higher CSF levels, failure in axonal repair	See also A
(2) Imaging		
RNFL thinning	+++ Axonal loss in the optic nerve	See also E, F
Hyperintense T2-weighted lesions	++ Combination of different mechanisms	See also A, C, E
Black holes	+++ Axonal loss	See also E
MTR decrease	++ Demyelination and axonal loss combined	See also C
DTI abnormalities	++ Combination of different mechanisms	See also C, E
MRS findings (especially NAA)	++	See also A, C, E
DTS	+++ Promising but still not widely accessible	See also C
Visual and motor EPs	++	See also A, C, D

(E) Prognostic biomarkers—biomarkers of disability progression (criteria ii, iv, v, vi, and viii)		

(1) Genetic-immunogenetic		
HLA-DRB1*1501	+/− Early progression from RRMS to SPMS	See also A, B
HLA-DRB1*1501	+ Worst brain atrophy measures	
HLA-DQB1*0301	+ Worst brain atrophy measures	
HLA-DQB1*0602	+ Worst whole and gray matter atrophy measures	
TOB1	+++ Early conversion from CIS to CDMS	See also C
(2) Laboratorial		
OCB IgG	+++ Conversion from CIS to CDMS	See also A
KFLC	+++ Conversion from CIS to CDMS	See also A
OCB IgM	+/− Bad prognostic biomarker	See also B
MRZ reaction	+++ Conversion from CIS to CDMS	See also A, F
Anti-MBP	+/− Conversion from CIS to CDMS	See also A, B
Anti-MOG	+/− Conversion from CIS to CDMS	See also A, B, F
AR	++ Marker of clinical severity in NMO	See also A, C
VEGF-A	++ Lower levels, progression from RRMS to SPMS	See also A, D
NO and NO metabolites	++ Higher CSF levels, longer relapses/higher disability progression rates	See also A, C
NF-H	+++ Higher CSF levels, progressive forms/bad prognostic biomarker	
NF-H and tau	+++ Combined high CSF levels, conversion from CIS to CDMS	
Tubulin/actin	++ Higher CSF levels, progressive forms/worst disability scores	
NAA	+++ Lower CSF levels, progressive forms/worst disability scores	See also A, D
GFAP	++ Higher CSF levels, progressive MS forms/worst disability scores +++ Disability progression in NMO	See also A,C
S100B	+ Disability progression in NMO	See also A,C
BDNF	++ Lower CSF levels in SPMS patients	See also C, D, F
Unblocked *α*4 integrin	+ Prognostic factor of risk for PML	See also C, F
(3) Imaging		
RNFL thinning	+ Correlation with brain atrophy measures and disease progression	See also D, F
Hyperintense T2-weighted lesions	+/−	See also A, C, D
Black holes	+/−	See also D
Whole brain atrophy measures	++ Worsening rates at MS onset, prognostic biomarker of disability after 8 years	
Gray matter atrophy measures	+++ Higher worsening rates, progressive forms/early CIS conversion to RRMS	
UCCA atrophy	++ Progressive forms, good correlation with EDSS, bad prognostic in RRMS	See also B
DTI abnormalities	+++ Early prognostic biomarker of relapse	See also C, D
Corpus callosum DTI abnormalities	+++ Bad prognostic biomarker	See also A
Spinal cord DTI abnormalities	+++ Good correlation with EDSS scores	
Early MRS abnormalities	++ Bad prognostic biomarker	See also A, C, D
Combined EPs	+++ Good prognostic biomarker, especially for benign disease forms	See also A, C, D
SSR	++ Correlation with higher EDSS scores	See also A

(F) Biomarkers of therapeutical response (criteria i, iv, v, vi, and vii)		

(1) Genetic-immunogenetic		
HLA-DRB1*0401, 0408, 1601	+++ Higher risk for developing neutralizing antibodies against IFN-B	See also A
(2) Laboratorial		
MRZ reaction	++ B-cell immunity targeted therapy	See also A, E
Anti-MOG	++ B-cell immunity targeted therapy	See also A, B, E
Fetuin-A	+++ Decreased CSF levels in Natalizumab responders	See also C
MBP	+++ Decrease in CSF levels in methylprednizolone responders	See also C
CSF lipocalin 2	++ Decreased CSF levels in Natalizumab responders	See also A
Unblocked *α*4 integrin	+++ Therapeutical response to Natalizumab	See also C, E
NF-L	+++ Normalized CSF levels in Natalizumab responders	See also A, C
BDNF	+++ CSF elevation in Glatiramer Acetate responders	See also C, D, E
TRAIL	++ Serum levels good predictors of response in IFN-B	
MxA	++ Serum levels good predictors of response in IFN-B	
sVCAM	++ CSF alterations in IFN-B responders	See also C
Th17 immune profil	+/− Immune response exacerbation by IFN-B	
Vitamin D	+++ Increased levels in IFN-B responders	See also A, C
sICAM-1	+ Lower levels in Cladribine responders	See also C
sE-Selectin	+ Lower levels in Cladribine responders	
(3) Imaging		
RNFL	+++ Biomarker of therapeutical efficacy for several agents	See also D, E

Classification of biomarkers. +++ very strong correlation, ++ strong correlation, + modest correlation, and +/− controversial correlation. Criteria used for classification., (i) Biological rationale; (ii) clinical rationale; (iii) predictability of disease initiation, reactivation or progression, or of disease differentiation; (iv) sensitivity and specificity; (v) reproducibility; (vi) practicality; (vii) correlation with therapeutical outcome; (viii) correlation with prognosis and disability. Biomarkers of more than one category are indicated in the third column.
